# Long-Term Follow-Up of Patients after Percutaneous Coronary Intervention with Everolimus-Eluting Bioresorbable Vascular Scaffold

**DOI:** 10.5935/abc.20160202

**Published:** 2017-02

**Authors:** Rafael Alexandre Meneguz-Moreno, José de Ribamar Costa Junior, Freddy Antônio Britto Moscoso, Rodolfo Staico, Luiz Fernando Leite Tanajura, Marinella Patrizia Centemero, Auréa Jacob Chaves, Andrea Claudia Leão de Sousa Abizaid, Amanda Guerra de Moraes Rego e Sousa, Alexandre Antonio Cunha Abizaid

**Affiliations:** Instituto Dante Pazzanese de Cardiologia, São Paulo, SP - Brazil

**Keywords:** Percutaneous Coronary Intervention, Absorbable Implants / utilization, Everolimus, Coronary Artery Disease, Clinical Evolution

## Abstract

**Background:**

Bioresorbable vascular scaffolds (BVS) were developed to improve the long-term results of percutaneous coronary intervention, restoring vasomotion.

**Objectives:**

To report very late follow-up of everolimus-eluting Absorb BVS (Abbott Vascular, Santa Clara, USA) in our center.

**Methods:**

Observational retrospective study, in a single Brazilian center, from August 2011 to October 2013, including 49 patients submitted to Absorb BVS implantation. Safety and efficacy outcomes were analyzed in the in-hospital and very late follow-up phases (> 2 years).

**Results:**

All 49 patients underwent a minimum follow-up of 2.5 years and a maximum of 4.6 years. Mean age was 56.8 ± 7.6 years, 71.4% of the patients were men, and 26.5% were diabetic. Regarding clinical presentation, the majority (94%) had stable angina or silent ischemia. Device success was achieved in 100% of cases with 96% overall procedure success rate. Major adverse cardiovascular events rate was 4% at 30 days, 8.2% at 1 year, and 12.2% at 2 years, and there were no more events until 4.6 years. There were 2 cases of thrombosis (1 subacute and 1 late).

**Conclusions:**

In this preliminary analysis, Absorb BVS showed to be a safe and effective device in the very late follow-up. Establishing the efficacy and safety profiles of these devices in more complex scenarios is necessary.

## Introduction

In the era of drug-eluting stents (DES), percutaneous coronary intervention (PCI) significantly improved clinical outcomes, with a reduction in excessive neointimal proliferation by adding antiproliferative agents. The permanent presence of intracoronary metal devices and long-lasting polymers, however, can delay natural vascular healing, resulting in constant inflammatory response and unfavorable clinical outcomes.^1-3^

Bioresorbable vascular scaffolds (BVS), thus, appeared as an alternative to those permanent prostheses: they can maintain the mechanical properties of metallic DES in the first months, and then be completely reabsorbed, eliminating possible adverse effects of their presence in the coronary arteries. 

Recently developed, the Absorb BVS (Abbot Vascular, Santa Clara, USA) is aimed at meeting the above-mentioned criteria, maintaining the efficacy profile of last-generation metallic DES. The Absorb BVS was assessed in humans for the first time in the ABSORB clinical trial (cohorts A and B), with promising results.^4-6^

Based on those results, the ABSORB EXTEND study, a multicenter single-arm study, has been conducted in 56 centers of several countries, aimed initially at including around 800 patients and at assessing the safety and performance of the Absorb BVS in a larger and more diversified population, as compared to that of initial studies, with more complex lesions.^7^

The present analysis reports the very late follow-up (>2 years) of the first patients submitted to Absorb BVS implantation in Brazil, as part of the ABSORB EXTEND multicenter registry.

## Methods

### Study design and target population

The present study included the patients treated with Absorb BVS between August/2011 and October/2013, in a tertiary cardiological center in Brazil, who were included in the international multicenter single-arm study, ABSORB EXTEND study, as part of the first 512 patients recruited in 56 centers of Europe, Australia, New Zealand, Japan, Hong Kong, Malaysia, Singapore, Latin America and Canada. 

It is worth noting that the participation in the EXTEND registry marks the beginning of the Brazilian experience with that new technology. This study was financed by Abbot Vascular, Santa Clara, USA. The Ethics Committee on Research of our institution approved the study protocol, and all patients provided written informed consent.

### Inclusion/exclusion criteria

Patients with the following characteristics were included in the study: age ≥ 18 years; evidence of myocardial ischemia, such as stable or unstable angina; silent ischemia; and functional test or transient alterations on 12-lead electrocardiography compatible with ischemia.

The patients had up to two *de novo* lesions that could be percutaneously treated, each located in separate native epicardial vessels. The lesions should be in a native coronary vessel, whose target-vessel diameter was ≥ 2.0 mm and ≤ 3.3 mm, and whose target-lesion extension was ≤ 28 mm, both assessed by use of on-line quantitative coronary angiography (QCA) or intracoronary ultrasound (ICUS). The target lesions should be in an artery or branch of significant caliber and stenosis should be visually estimated ≥ 50% and < 100%, with TIMI (*Thrombolysis in Myocardial Infarction*) flow ≥ 1. Previous PCI in a non-target vessel was allowed, if performed at least 30 days after the index procedure or planned for 6 months after the index procedure; PCI in target-vessel lesions were allowed if performed at least 6 months before the index procedure or planned to 6 months after the index procedure.

Patients with the following characteristics were excluded from the study: previous acute myocardial infarction (AMI) up to 3 days before the index procedure; arrhythmias with hemodynamic instability; left ventricular ejection fraction < 30%; chronic renal failure; left main coronary artery lesions; lesions in arterial or venous grafts; in-stent restenosis; bifurcation lesions; total occlusion (TIMI flow 0); and significant calcification or excessive tortuosity.

### Device

We used the Absorb BVS, the same device used in cohort B of the ABSORB study.^8,9^ The Absorb platform is composed by the polymer poly-L-lactic acid (PLLA), the antiproliferative drug everolimus (Novartis Pharmaceuticals Corporation, Basel, Switzerland), and a matrix of poly-D, L-lactic acid (PDLLA), at a 1:1 ratio, forming an amorphous matrix covered with 100*µ* everolimus/cm^2^. Both PLLA and PDLLA are metabolized and resorbed in the body. PDLLA is expected to be completely resorbed by the arteries in 9 months, while PLLA, in approximately 36 months. During resorption, the chains with PLLA and PDLLA are hydrolyzed, the last product of that reaction being lactic acid, biologically metabolized via Krebs cycle.^5^

At the time the patients were included in this study, Absorb devices were available only in two diameters (2.5 and 3.0 mm) and two lengths (18 and 28 mm).

### Procedure

All procedures were performed electively, in accordance with current guidelines. The lesions were treated with the usual intervention techniques, which required pre-dilatation with a shorter balloon, with a diameter 0.5 mm smaller than that of the device used. The Absorb's deployment pressure should never exceed the manufacturer's maximum nominal reference value. 

Post-dilatation was subjected to need and operator's assessment. It was performed with non-compliant balloons, within the expansion limits of the BVS (post-dilatation balloons should not exceed 0.5 mm the nominal diameter of the implanted BVS).

Preprocedural dual antiplatelet therapy comprised an attack dose of acetylsalicylic acid (300 mg) and clopidogrel (300 mg), at least 24 hours before the procedure, or 600 mg if < 24 hours. After the intervention, acetylsalicylic acid was prescribed indefinitely and clopidogrel (75 mg/day) was maintained for at least 6 months.

### Quantitative coronary angiography and intracoronary ultrasound

The recommended limits of the target-vessel's diameter were established by use of on-line QCA on distal and proximal maximal luminal diameter (Dmax), the Dmax being assessed in the distal and proximal portions of the target segment to be coated with the BVS, or by use of ICUS. Overlapping of the BVS was allowed for lesions > 22 mm and ≤ 28 mm, with a recommended limit of 1-4 mm.

### Follow-up

Clinical follow-up, via outpatient clinic consultation or telephone, was mandatory at day 30 (± 7 days), 6 months (± 14 days) and 1, 2 and 3 years (± 28 days), following the ABSORB EXTEND study protocol. After that, routine return visits were recommended. Minimum follow-up was 2.5 years. All adverse events and symptoms, such as angina, details of subsequent PCIs, as well as medication use and changes, were collected in the period. The patients did not undergo a new protocol coronary angiography, being only reassessed in case of clinical indication due to symptoms or evidence of ischemia.

### Study outcomes

All outcomes were adjudicated by an independent clinical events committee abiding by the protocol definitions based on the Academic Research Consortium (ARC).^10^

Clinical success comprised device's success (based on the target lesion) and procedural success (assessed in each patient). In addition, it included scaffold thrombosis (ST), cardiovascular death, AMI (either related or not to the target vessel) and revascularization rate (target-lesion or target-vessel revascularization, or total revascularization). In addition, combined outcome rates, considering ischemia-driven (ID) major adverse cardiovascular events (MACE) (ID-MACE), ID target-vessel failure (ID-TVF), ID target-vessel revascularization and ID target-lesion revascularization (ID-TLR), were assessed.

The device's success was defined as successful device's deployment in the target lesion and successful withdrawal of the BVS delivery system, with residual stenosis < 50% assessed via QCA (or visual estimate, when QCA was unavailable).

The procedure's success was defined as device's success with no ID-MACE during hospitalization for up to 7 days after the procedure. If there were two lesions, both should meet the success criteria.

Cardiac death was defined as any death of cardiac cause, such as AMI, low output syndrome, and lethal arrhythmia. Unattended death and death of unknown cause were classified as cardiac death. This included the deaths related to the procedure.

The classification of AMI and the diagnostic criteria were defined based on the pre-established protocol:^11^ Q-wave AMI, characterized by the development of a new pathological Q wave; Non-Q-wave AMI, defined as elevation of creatine phosphokinase (CK) levels ≥ 2 times the upper limit of normality with concomitant increase in CK-MB in the absence of new pathological Q waves.

The revascularization events were defined as follows:

- ID-MACE: composed of cardiac death, Q-wave/non-Q-wave AMI, target-lesion revascularization via PCI or coronary artery bypass graft (CABG);
- ID-TVF: composed of cardiac death, AMI with and without Q wave, target-vessel revascularization via PCI or CABG;
- ID-TLR: defined as any new PCI in the target lesion, either percutaneous or CABG in the target vessel with positive functional ischemia, ischemic symptoms or angiography evidencing lumen diameter at stenosis ≥ 50% by use of QCA, or revascularization of a target lesion with diameter ≥ 70% by use of QCA without ischemic symptoms or functional test.

Scaffold thrombosis was categorized as acute (< 1 day), subacute (1-30 days), late (> 30 days and < 1 year) and very late (>1 year), and defined based on the ARC guidelines as follows:^10^ definite (acute coronary syndrome and pathological or angiographic confirmation of the BVS thrombosis) or likely (death of unknown cause ≤ 30 days or AMI related to the target vessel without angiographic confirmation).

### Statistical analysis

Continuous variables with normal distribution were expressed as mean and standard deviation. Categorical variables were expressed as absolute numbers and percentages. The SPSS program (Statistical Package for the Social Science, Chicago, USA), version 19, was used for data tabulation.

## Results

The present study represents the analysis of 49 patients (53 lesions/57 BVS) included in the ABSORB EXTEND study and submitted to PCI with Absorb BVS implantation, at a Brazilian tertiary cardiology center. Clinical 1-year follow-up was obtained in 100% of the cases, while 2-year follow-up, in 97.9% of the cases. Mean follow-up was 3.59 ± 0.72 years (2.5-4.6 years).

Table 1 shows the demographic and clinical characteristics of the population studied. The patients' mean age was 56.8 ± 7.6 years, most of them were men (71.4%), and 26.5% of the population studied had diabetes. In addition, only 6.1% of the patients had more than one target lesion, and 6.1% of the patients presented with clinical findings of acute coronary syndrome (55.1%, stable angina; 38.8%, silent ischemia). Neither ST-segment elevation AMI nor recent AMI occurred.

**Table 1:** Demographic and clinical characteristics

	ABSORB BVS (n = 49)
Age (years), mean	56.8 ± 7.6
Male sex, n (%)	35 (71.4)
Diabetes, n (%)	13 (26.5)
Insulin-dependent diabetes mellitus, n (%)	5 (10.2%)
Hypertension, n (%).	39 (79.6)
Dyslipidemia, n (%)	38 (77.6)
Smoking, n (%)	30 (6.1)
Renal failure (CrCl < 60 mL.min), n (%)	0
Peripheral vascular disease, n (%)	4 (8.1)
Previous AMI, n (%)	30 (61.2)
Previous PCI, n (%)	30 (6.1)
Previous CABG, n (%)	2 (4.1)
Clinical presentation, n (%)	
Stable angina	27 (55.1)
NSTEACS	3 (6.1)
Silent ischemia	19 (38.8)

CrCl: creatinine clearance; AMI: acute myocardial infarction; PCI: percutaneous coronary intervention; CABG: coronary artery bypass graft; NSTEACS: Non-ST segment elevation acute coronary syndrome.

Table 2 illustrates the angiographic characteristics of the lesions treated and the procedure. Most lesions treated were in the anterior descending coronary artery (46.9%), followed by the right coronary (32.6%) and circumflex (26.5%) arteries. The mean grade of stenosis was 76.0 ± 8.5%. By use of on-line QCA or ICUS, the lesions had a mean diameter of 2.92 ± 0.28 mm (range, 2.2-3.5 mm) and a mean extension of 15.98 ± 5.55 mm (range, 7-28 mm).

**Table 2:** Angiographic and procedural characteristics

	ABSORB BVS (n = 49)
**Target vessel, n (%)**	
Anterior descending coronary artery	23 (46.9)
Right coronary artery	16 (32.6)
Circumflex artery	13 (26.5)
Multiple vessels	6 (12.2)
Diameter of the lesion, mm	2.92 ± 0.28
Length of the lesion, mm	15.98 ± 5.55
Mean grade of stenosis, (%)	76.0 ± 8.5
**Number of target lesions, n (%)**	
One	39 (93.9)
Two	3 (6.1)
Pre-dilatation, n (%)	49 (100)
Post-dilatation, n (%)	46 (93.8)
Angiographic success, n (%)	49 (100)
Device success, n (%)	49 (100)
Procedural success, n (%)	47 (95.9)

The device's clinical success was 100%, while the procedure's clinical success was 96% (47/49) in the 49 patients submitted to PCI with Absorb implantation. Two patients (4%) had periprocedural AMI while hospitalized.

Table 3 shows the clinical outcome data at 30 days and 1 year, and the very late follow-up of the patients. At 30 days, the MACE rate was 4% because of the periprocedural AMI rate. Cardiac mortality, target-vessel revascularization and non-target-vessel revascularization was 0%.

**Table 3:** Clinical outcomes in early, middle-term and long-term follow-up

	30 days n = 49	12 months n = 49	24 months n = 48	36 months n = 30	48 months n = 16
MACE, n (%)	2 (4)	4 (8.2)	6 (12.2)	6 (12.2)	6 (12.2)
Global mortality, n (%)	0	1 (2)	1 (2)	1 (2)	1 (2)
Cardiac death, n (%)	0	1 (2)	1 (2)	1 (2)	1 (2)
**AMI, n (%)**					
Q-wave AMI	0	0	0	0	0
Non-Q-wave AMI	0	0	1 (2)	1 (2)	1 (2)
Periprocedural AMI, n (%)	2 (4)	-	-	-	-
ID-target-vessel revascularization, n (%)	0	1 (2)	1 (2)	1 (2)	1 (2)
ID-target-lesion revascularization, n (%)	0	0	1 (2)	1 (2)	1 (2)
MR not related to ID-target-vessel or lesion, n (%)	0	0	1 (2)	2 (4)	2 (4)
**Scaffold thrombosis, n (%)**					
Acute	0	-	-	-	-
Subacute	1	-	-	-	-
Late	-	1	-	-	-
Very late	-	-	0	0	0
Stroke, n (%)	0	0	0	0	0

MACE: major adverse cardiovascular events; AMI: acute myocardial infarction; MR: myocardial revascularization; ID: ischemia directed.

At 1 year, the MACE rate was 8.2%, because of cardiac death and need for revascularization of the target vessel (but not of the target lesion) via PCI in one patient, the global AMI rate being maintained as 0%. At 2 years, the MACE rate was 12.2% because of a non-Q AMI event related to the target vessel and one in-stent restenosis event requiring target-lesion revascularization. From 2 years of follow-up till now, there were neither cardiovascular nor cerebrovascular events, and the accumulated MACE rate remained as 12.2% among the patients followed up till almost 5 years.

Regarding device's thrombosis and based on the ARC criteria, the findings were as follows: one case of definite subacute thrombosis 13 days after implantation, need for urgent surgical vascular procedure and a new angiographic study with an unsuccessful recanalization attempt; and one case of likely late thrombosis 34 days after PCI (sudden death episode). After one year, there was no additional case of thrombosis. 

## Discussion

In this initial experience, at a single center, the Absorb BVS performed well in the long run, with a very low target-vessel failure rate.

In the past 3 years, more than 60,000 patients were treated with Absorb BVS worldwide, despite the lack of a robust randomized study comparing it with contemporary drug-eluting stents.^12^

The assessment of Absorb BVS has begun with the ABSORB cohort studies A and B and clinical trial.^13,14^ After changes in the device's design and structure, the device's current version began to be used in cohort B, involving 101 patients, and showed a 1-year late lumen loss of 0.27 mm, the 2-year follow-up evidencing a MACE rate of 6.8% and no device's thrombosis.^5,15^ At 5 years, the Absorb's structures were no longer discernible on optical tomography or ICUS, the MACE rate being 11%, with no evidence of thrombosis.^16^

The initial analysis of the first 512 patients recruited in the ABSORB EXTEND registry, in a 1-year follow-up, confirms the efficacy of Absorb BVS, with very low incidence of ID-MACE (4.8%), ID-TVF (4.4%) and device's thrombosis (0.8%).^7^ At 3 years, with 250 patients, the MACE rate was 9.3%, the ID-TVF, 10.1%, and thrombosis, 1.2%.^17^

In our study, the MACE rate in a very late follow-up was equivalent, with no event after 2 years, corroborating the theory that the major benefit of the BVS occurs in the long run, with both low rate of events and the likelihood of new revascularization and BVS assessment by use of non-invasive imaging techniques.

Regarding the comparison with the results of drug-eluting metal stents, no long-term follow-up study has been published. In a recent meta-analysis encompassing the last four randomized studies comparing Absorb BVS with the everolimus-eluting metal stent Xience® (Abbot Vascular, Santa Clara, USA), ABSORB II,^18^ ABSORB III,^19^ ABSORB Japan^20^ and ABSORB China,^21^ the relative combined outcomes rates at the end of the first year did not differ between the Absorb and Xience groups (11.9% vs. 10.6%, respectively, p=0.38). Target-vessel AMI was significantly higher in the Absorb group as compared to the Xience group (5.1% vs. 3.3%, respectively, p=0.04), due partially to the higher rate of periprocedural AMI and partially to the higher rate of ST (definite or likely) in the Absorb group (1.3% vs. 0.6%, respectively, p=0.08). The results were similar after multivariate analysis adjusted to baseline characteristics, and were consistent even in the analysis of most subgroups.^22^

The EVERBIO-II Trial (*Comparison of Everolimus- and Biolimus-Eluting Stents With Everolimus-Eluting Bioresorbable Vascular Scaffold Stents II*), a single-center study, involved 240 patients randomized at the 1:1:1 proportion for everolimus-eluting stent, biolimus-eluting stent or Absorb BVS. In a 2-year follow-up, the MACE rate related to the device was 13% in the everolimus- and biolimus-eluting stent groups vs. 21% in the Absorb group (p=0.12), and the related MACE rate was 32% vs. 35%, respectively (p=0.67), with only one ST event in the Absorb group and none in the DES groups (p=0.33). Thus, once again DES were considered non-inferior to BVS.^23,24^

Regarding other BVSs, the DESolve® NX (Elixir Medical Corporation, Sunnyvale, USA) was the only BVS with late follow-up and recently published results. At 2 years, that new device showed the following rates: MACE, 7.4%; isolate cardiac death, 2.5%; AMI, 0.8%; target-lesion revascularization, 4.1%; and target-lesion failure, 7.4%. In addition, the thrombosis rate was minimal (0.8%).^25^

Tamburino et al., using a complex statistical analysis, have assessed the database of the GHOST-EU Registry (*Gauging coronary Healing with biOresorbable Scaffolding plaTforms in EUrope*), with 1,189 patients treated with Absorb BVS in Europe and 5,034 patients treated with everolimus-eluting metal stent (Xience) of the XIENCE V Registry in the USA. After propensity score matching, 905 pairs of patients were identified with similar characteristics. Of the total of 1,810 patients, there was no difference between the Absorb and Xience groups concerning the risk of MACE within 1 year (5.8% vs. 7.6%, respectively, p=0.12). Cardiac death was less likely to occur in the Absorb group (0.7% vs. 1.9%, p=0.03) and there was a tendency towards reduction in AMI in the Absorb group as compared to the Xience group (2.4% vs. 4.0%, p=0.07). In addition, there was no difference in target-vessel revascularization (4.6% vs. 3.5%, p=0.22) and definite or likely thrombosis (1.8% vs. 1.1%) between the Absorb and Xience groups, respectively.^26^ In most studies, the ST cases occurred in the immediate post-procedural period (<30 days), and cases after the sixth month were rare, as observed in the cohort reported. 

### Limitations

This was a retrospective and observational study, having, thus, obvious limitations. The sample was small, with low clinical and anatomical complexity, following the ABSORB EXTEND study protocol. 

## Conclusions

In this case series, Absorb BVS implantation was associated with a low incidence of adverse events, mainly in the very long-term follow-up (> 2 years). However, larger studies with a higher number of patients and more complex scenarios are necessary to confirm these preliminary observations.
